# Effects of Flavonoids on Rumen Fermentation Activity, Methane Production, and Microbial Population

**DOI:** 10.1155/2013/349129

**Published:** 2013-09-24

**Authors:** Ehsan Oskoueian, Norhani Abdullah, Armin Oskoueian

**Affiliations:** ^1^Institute of Tropical Agriculture, Universiti Putra Malaysia, 43400 Serdang, Selangor, Malaysia; ^2^Agriculture Biotechnology Research Institute of Iran (ABRII), East and North-East Branch, P.O. Box 91735/844, Mashhad, Iran; ^3^Department of Biochemistry, Faculty of Biotechnology and Biomolecular Sciences, Universiti Putra Malaysia, 43400 Serdang, Selangor, Malaysia; ^4^Ferdowsi University of Mashhad, International Branch, P.O. Box 91779/4888, Mashhad, Iran

## Abstract

This research was carried out to evaluate the effects of flavone, myricetin, naringin, catechin, rutin, quercetin, and kaempferol at the concentration of 4.5% of the substrate (dry matter basis) on the rumen microbial activity *in vitro*. Mixture of guinea grass and concentrate (60 : 40) was used as the substrate. The results showed that all the flavonoids except naringin and quercetin significantly (*P* < 0.05) decreased the dry matter degradability. The gas production significantly (*P* < 0.05) decreased by flavone, myricetin, and kaempferol, whereas naringin, rutin, and quercetin significantly (*P* < 0.05) increased the gas production. The flavonoids suppressed methane production significantly (*P* < 0.05). The total VFA concentration significantly (*P* < 0.05) decreased in the presence of flavone, myricetin, and kaempferol. All flavonoids except naringin and quercetin significantly (*P* < 0.05) reduced the carboxymethyl cellulase, filter paperase, xylanase, and **β**-glucosidase activities, purine content, and the efficiency of microbial protein synthesis. Flavone, myricetin, catechin, rutin, and kaempferol significantly (*P* < 0.05) reduced the population of rumen microbes. Total populations of protozoa and methanogens were significantly (*P* < 0.05) suppressed by naringin and quercetin. The results of this research demonstrated that naringin and quercetin at the concentration of 4.5% of the substrate (dry matter basis) were potential metabolites to suppress methane production without any negative effects on rumen microbial fermentation.

## 1. Introduction

The highly diverse methanogenic community present in the rumen have been implicated in global warming, and attempts to manipulate rumen microbial fermentation towards reducing the methane production through application of feed additives remain a high priority [1]. For the past decades, several additives, such as ionophores and probiotics, have been introduced to the ruminant production industry [2]. The ionophores such as monensin, lasalocid, and laidlomycin significantly suppressed the methane production in ruminants [3]. However, concerns including antibiotic resistance and detectable residual levels of these compounds in animal products limit the utilization of these additives [4]. In the case of probiotics, the commonly used microorganisms for ruminants are yeast and *Aspergillus oryzae.* These microbes increase butyrate or propionate acids concentration, reduce protozoa numbers, and promote acetogenesis which resulted in lower methane production [5]. However, the use of probiotics to inhibit methane production in ruminants is limited due to the cost; hence, appropriate strategies are required for the large-scale production of probiotics with economical operating expenditure [6].

Recently, natural plant products which are often inexpensive and environmentally safe have been introduced in methane mitigation strategies. They could be superior feed additives to replace the ionophores and probiotics for controlling methanogenesis [7]. These compounds are not only able to suppress the methane production but also possess broad range of favorable effects on animal health. For instance, their major affects on gastrointestinal tract include improvement in digestibility, feed efficiency, protection of dietary proteins from rumen microbial degradation, maintaining the gut microflora balance, gastric or liver damage prevention, reduction in gastrointestinal spasms, diarrhea, constipation, bloat, acidosis, and controlling gut pathogens [8].

Furthermore, their main effects on respiratory and cardiovascular system in animals include emollient, antitussive, expectorant, hypotensive, cardioprotective, and vascular-stabilizing properties [9]. They also possess antihyperlipidemic, hypocholesterolaemic, and diuretic properties. These compounds are capable of reducing fear, depression, and anxiety and showed to have antipyretic and analgaesic effects. The enhancements in immune function, reproductive organs, fertility, wool growth, and ectoparasites elimination have also been reported [8, 10].

Among plant secondary metabolites, flavonoids have gained importance because of their wide range of biological activities and in particular antimicrobial properties. Flavonoids are classified under polyphenolic compounds as they possess A and C rings of benzo-1-pyran-4-quinone and a B ring [11]. These natural compounds are believed to have direct effects against methanogens [12] and to be an alternative agent to suppress methane production and improve animal health and productivity.

The plant flavonoids are generally present in the glycosides form with the aglycone linked to a variable sugar moiety by a *β*-glycosidic bond, mainly in position 3 of the C ring [11, 13]. The presence of sugar moiety reduces the bioactivity of flavonoid; thus, the removal of sugar moiety not only enhances the functional properties of flavonoid but also improves the bioavailability in the gastrointestinal tract. A recent study by Berger et al. [13] showed that rumen microbes enhanced the bioavailability of flavonoid rutin (quercetin-3-O-rutinoside) by degradation of glycosidic linkage. This degradation resulted in libration of quercetin. Further, the quercetin and its methylated (isorhamnetin, tamarixetin) and dehydroxylated (kaempferol) derivatives were detected in plasma of nonlactating cows. Similarly, a recent study by Gohlke et al. [14] compared the bioavailability of quercetin in the aglycone and glucorhamnoside forms through duodenal administration in German Holstein cows. Their results showed higher intestinal bioavailability of quercetin in the aglycone form as compared to the glucorhamnoside form.

Plant extracts rich in flavonoids have gained importance in improving animal production. Tedesco et al. [15] reported the increase in milk yield and lactation performance in dairy cows upon 25 d administration of sylimarin (10 g/d) which mainly consist of flavonolignans. Likewise, Balcells et al. [16] showed that plant extract containing flavonoids at the concentration of 300 mg/kg DM was able to decrease the incidence of acidosis and enhance the animal growth performance in cattle receiving high-concentrate diet. This phenomenon was attributed to the decrease in the titers of *Streptococcus bovis* and *Selenomonas ruminantium *and increase in the numbers of lactate-consuming microorganisms such as *Megasphaera elsdenii*. 

Currently, various flavonoids-rich feed additives to suppress the methane production are available in the market. However, these products mainly contain plant crude extracts, and it is rather difficult to correlate the response of rumen microbes to the flavonoids. The presence of other components such as glycosides, phenolics, terpenoids, alkaloids, essential oils, and organic acids in the plant extracts may influence the results. Furthermore, the information on the effect of flavonoids in the pure form on rumen microbial activity is still lacking [13, 14, 16]. 

Taking all these considerations into account, this research hypothesised that the flavonoids depending on their types are capable of modulating the rumen fermentation activity in varying degrees. Hence, in order to test this hypothesis, *in vitro* gas production technique was applied to evaluate the effect of different types of flavonoids in the pure forms on rumen microbial fermentation, methane production, enzyme activity, microbial protein synthesis, and microbial population.

## 2. Material and Methods

### 2.1. Flavonoids

The flavonoids (purity ≥ 98%) consisting of flavone, myricetin, naringin, catechin, rutin, quercetin, and kaempferol were purchased from Sigma (St. Louis, MO, USA).

### 2.2. *In Vitro* Rumen Fermentation

The *in vitro* gas production technique has been considered as an acceptable method to evaluate the effect of phytochemicals on rumen microbial fermentation [17]. Two male cows, fitted with rumen fistula, were maintained on a diet consisting of 60% guinea grass and 40% commercial cow pellet (FFM Berhad, Malaysia) which contained corn grain, palm kernel cake, soybean meal, rice bran, palm kernel oil, limestone, salt, and vitamin-mineral premix. The proximate chemical composition of the diet (g/kg DM) was 146 crude protein, 485 neutral detergent fiber, 36.9 crude lipid, 83.9 ash, and metabolizable energy 10.04 MJ/Kg DM. The diet was offered twice daily and animals had free access to drinking water. The animals were used as rumen fluid donors. 

Two hundred milligram of feed consisting of dry guinea grass and concentrate at 60 : 40 ratio was used as the substrate for the *in vitro* fermentation. The incubation medium was prepared as described by Menke and Steingass [18], and 30 mL was dispensed anaerobically into each 100 mL syringe. Each flavonoid was dissolved in ethanol and the concentration of 4.5% (w/w) of the substrate on dry matter basis (9 mg) was included in each syringe. The final ethanol concentration of each syringe was 0.5% (v/v). The control consisted of substrate with 0.5% (v/v) ethanol. The syringes were incubated at 39°C for 24 h. *In vitro* gas production (GP) was measured at 2, 4, 8, 12, and 24 h. A total of nine syringes for each treatment were used. The content of three syringes were used for dry matter degradability (DMD), pH, and fermentation parameters and another three for microbial protein synthesis, and the remaining three syringes were used for quantification of rumen microbial population and enzyme activity assays. This experiment was performed in three separate runs. The volatile fatty acids (VFAs), which include acetic, isobutyric, butyric, propionic, valeric, isovaleric, and caproic acids, were determined by gas chromatography (Agilent 6890 A) which was equipped with a capillary column packed with 10% (w/v) PEG 600 on Shimalate TPA 60/80 [19]. After 24 h incubation, methane production was measured by injecting 1 mL of the headspace gas from each of the syringes into a gas chromatograph (Agilent 5890 Series Gas Chromatograph, Wilmington, DE, USA) equipped with FID detector. Separation was achieved using an HP-Plot Q column (30 m × 0.53 mm × 40 m) (Agilent Technologies, Wilmington, DE, USA) with nitrogen (99.9% purity, Domnick-Hunter generator, Domnick-Hunter, Leicester, UK) as the carrier gas at the flow rate of 3.5 mL/min. An isothermal oven temperature of 50°C was used in the separation. Calibration was completed using standard methane prepared by Scotty Specialty Gases (Supelco, Bellefonte, PA, USA). The ammonia nitrogen content was determined by the Kjeldahl procedure [20]. Cumulative gas production data were fitted to the model of Ørskov and McDonald [21], and the values of *a* (the gas production from the immediately soluble fraction), *b* (the gas production from the insoluble fraction), *a* + *b* (potential extent of gas production), and *c* (gas production rate constant for the insoluble fraction *b*) were estimated using the nonlinear regression (NLIN) procedure of SAS [22]. All animal management and sampling procedures were approved by the Universiti Putra Malaysia Animal Care and Use Committee [23].

### 2.3. Rumen Microbial Enzyme Activity

In order to extract the microbial enzyme, the whole content of each syringe after 24 h incubation was transferred to a 50 mL centrifuge tube and mixed with 5 mL carbon tetrachloride and lysozyme solution (0.4 g/100 mL phosphate buffer, 0.1 M, and pH 6.8) and further incubated at 40°C for 3 h followed by 60 s sonication at 4°C using a sonicator (Vibra Cell sonicator, Sonics and Materials, Danbury, CT, USA). The sonicated samples were centrifuged at 24,000 ×g for 20 min at 4°C, and the clear supernatant was used for the estimation of enzyme activities [24].

The enzymes studied were filter paperase (FPase), carboxymethylcellulase (CMCase), *β*-glucosidase, and xylanase as described by Saad et al. [25]. Filterpaper, carboxymethylcellulose, *ρ*-nitrophenyl-*β*-D-glucopyranoside, and xylan were used as substrates to determine the FPase, CMCase, *β*-glucosidase, and xylanase activities, respectively. Filterpaperase, CMCase, and xylanase activities were determined by measuring the production of reducing sugar using dinitrosalicylic acid (DNSA) [26]. *β*-glucosidase activity was measured by the amount of *ρ*-nitrophenol released from the *ρ*-nitrophenyl-*β*-D-glucopyranoside (PNPG). Each enzyme assay was carried out in triplicate. Protein content of supernatant was determined according to Bradford [27]. The specific activity of each enzyme (CMCase, FPase, xylanase, or *β*-glucosidase) was expressed as *μ*mol of product (glucose/xylose/4-nitrophenol) released/min/mg protein under the assay conditions.

### 2.4. Rumen Microbial Protein Synthesis

Microbial protein synthesis was determined according to the method described by Makkar and Becker [28] using purines as a marker. After 24 h fermentation, the content of each syringe was centrifuged at 20,000 ×g for 30 min and the supernatant was discarded. The pellet was washed with distilled water followed by centrifugation (20,000 ×g for 30 min). The pellet, consisting of undigested substrate and microbial mass, was lyophilized. Aliquot of 2.5 mL of perchloric acid (0.6 M) was added to 100 mg of each lyophilized sample and the mixture was incubated in a water bath at 90–95°C for 1 h. The pH of solution was adjusted between 6.6 and 6.9 using concentrated KOH (8 M) and the solution was centrifuged at 3,000 ×g for 15 min to remove the precipitate. Then, the supernatant was filtered through 0.45 *μ*m filter. The adenine and guanine contents were quantitatively measured in the supernatant by high-performance liquid chromatography (HPLC) equipped with a reverse phase C18 LiChrospher 100, 250 × 4 mm I.D and 5 *μ*m pore size column (Agilent Technologies, Waldbronn, Germany). Absorbance was monitored at 254 nm and guanine and adenine peaks appeared at about 8.3 and 11.1 min, respectively. Allopurinol was used as the internal standard which appeared at about 16.6 min. The efficiency of microbial protein synthesis (EMPS) was calculated by dividing the total purines by total gas or total volatile fatty acids (VFAs). 

### 2.5. Rumen Microbial Population Analysis

At the end of the incubation, 1 mL of rumen fluid containing digesta was used for DNA extraction using QIAamp DNA Stool Mini Kit (QIAGEN). The primer sets used in this study are shown in Table 1. The 16S rRNA of bacteria and 18S rRNA of protozoa, and fungi were amplified by PCR using primers for general bacteria, general fungi, total protozoa, *Ruminococcus flavefaciens, Fibrobacter succinogenes*, *Ruminococcus albus,* and total methanogens. The PCR products were cloned in pCR2.1-TOPO TA cloning vector (Invitrogen, Carlsbad, CA, USA) and transformed into chemically competent *E. coli* TOP10 cells (Invitrogen). The plasmids were extracted and sequenced using capillary electrophoresis on an Applied Biosystems 3730xl DNA Analyzer (Applied Biosystems, Foster City, CA, USA). The sequences were checked for chimeric rDNA using Bellerophon [29] and were compared to those available in the GenBank using the Basic Local Alignment Search Tool [30]. The plasmid carrying the sequence that was ≥99% similar to the previously published sequence of the target microorganism was used for real-time PCR amplification and standard curve construction. The concentration and purity of the plasmid for each group of microorganisms was determined using Nanodrop (NanoDrop Technologies, Wilmington, DE, USA), and the number of copies was determined using the following formula [31]

(1) Amount  of  DNA  (μg/mL)×6.022×1023Length(bp)×109×650.

Real-time PCR assays were conducted on a BioRad CFX 96 real-time PCR thermocycler (Bio-Rad, Hercules, USA) using iQ SYBR Green Supermix (Bio-Rad Laboratories, Inc., Hercules, CA, USA). Data from the real-time PCR reactions were analyzed using CFX manager software version 3 (Bio-Rad Laboratories). All real-time PCR amplifications were performed in triplicate.

### 2.6. Statistical Analyses

The data were analysed using the general linear models (GLM) procedure of SAS [22] in a completely randomized design (CRD), and means were compared with Duncan's multiple range test. Means were considered significantly different at *P* < 0.05.

## 3. Results and Discussion

The effects of flavonoids at the concentration of 4.5% (w/w) of the substrate on rumen dry matter (DM) degradability, total gas, and methane gas production kinetics are shown in Table 2. The *in vitro* DM degradability of control group was 87.9% and all flavonoids except naringin and quercetin reduced this value significantly (*P* < 0.05) to the range of 81.3 to 83.2%. The total gas production of the control was 36.1 mL (Table 2) and this value was significantly (*P* < 0.05) decreased to 28.1 and 30.6 mL when the flavone and myricetin were added, respectively. On the other hand, naringin, rutin, and quercetin increased the gas production significantly (*P* < 0.05) to 47.8, 40.9, and 43.0 mL, respectively. 

The control treatment showed the production of 8.6 mL/g DM methane and inclusion of flavone, myricetin, naringin, rutin, quercetin, and kaempferol significantly (*P* < 0.05) decreased the values to 5.7, 4.9, 6.3, 7.2, 6.2, and 5.3 mL/g DM, respectively. The inhibitory activities of flavonoids used in this experiment towards methanogenesis can be categorized in descending order as follows: myricetin ≥ kaempferol ≥ flavone > quercetin ≥ naringin > rutin ≥ catechin. The suppression of methane production observed in this study was in accordance with the result of Tavendale et al. [32] who demonstrated the potential of flavonol to decrease methane production in *Methanobrevibacter ruminantium* culture. Besides, Patra et al. [24] have also indicated that plant extract containing flavonoids could decrease the methane production. Generally, the decrease in the dry matter degradability, total gas, and methane production upon addition of flavonoids could be attributed to the antimicrobial action of flavonoids [33, 34].

The potential extent of gas production indicated by the *a + b* values is in accordance with the results in gas production during fermentation. As observed, these values were significantly (*P* < 0.05) higher in treatments with naringin, rutin, and quercetin and lower in treatments with flavone and myricetin (Table 2). The gas production rate constants for the insoluble fraction (*b*) are presented as *c* values in Table 2. The *c* value for the control was 0.08%, and addition of naringin, rutin, and quercetin reduced this significantly (*P* < 0.05). The increase in the gas production of *a + b* led to the decrease in the *c* value as previously described by Ørskov and McDonald [21].

The addition of flavonoids did not affect the pH and ammonia nitrogen significantly as shown in Table 3. The total VFA concentration of control group was 47.3 mM, but the addition of flavone, myricetin, and kaempferol significantly (*P* < 0.05) reduced the total VFA concentration to 41.3, 39.1, and 42.3 mM, respectively. The decrease in total VFAs values implied the antimicrobial action of flavonoids. However, in treatments with naringin, catechin, rutin, and quercetin, the total VFAs concentrations were comparable to the control. In the case of catechin and rutin, in spite of the decrease in DM degradability, the VFA concentration was not significantly suppressed, which indicated the possible utilization of these flavonoids as fermentable substrates. It has been reported by McSweeney et al. [35] that rutin, naringin, and quercitrin are readily degraded in the rumen and their derivatives are utilized by rumen microbes. Smith et al. [36] reported the microbial degradation of flavonoids in the rumen which occurred through cleavage of their C rings resulting in phenolic acids and nonaromatic fermentation products. Thus, these byproducts could play a role as an alternative carbon source for rumen microbial activities. 

The molar percentage of acetic acid and propionic acid were significantly (*P* < 0.05) reduced in treatments with flavone, myricetin, and kaempferol, with concomitant increase in butyric acid when compared to the control. On the other hand, molar percentages of acetic, propionic, and butyric acids in treatments with naringin, catechin, rutin, and quercetin were comparable to the control. In line with this result, Lowry and Kennedy [37] and McSweeney and Mackie [38] have reported the increase in concentration of acetic and butyric acids upon fermentation of rutin, naringin, and quercetin by rumen microbes. The increase in acetic to propionic (C2 : C3) ratio reflects an increase in acetic acid and slight decrease in propionic acid concentrations.

It is interesting to note that CMCase, FPase, xylanase, and *β*-glucosidase activities in treatments with naringin and quercetin were comparable to the control (Table 4), whereas other flavonoids reduced these activities significantly (*P* < 0.05). The results showed that the specific activity of xylanase in buffered rumen fluid was higher than that of the CMCase and FPase. Xylanase is a measure of hemicellulase activity, while CMCase and FPase indicate cellulolytic activity. The levels of enzyme activities were in accordance with the percentage of DM degradability. 

The decrease in CMCase, FPase, xylanase, and *β*-glucosidase specific activities of fermenting rumen fluid in the presence of flavone, myricetin, and kaempferol could be related to the higher antimicrobial action of these compounds or their derivatives produced during fermentation. The enzyme activities of rumen microbes treated with naringin and quercetin are in accordance with the results in DM degradability and end products of fermentation. The effects of naringin and quercetin on rumen fermentation in this research are similar to that of methanolic extract of garlic reported by Kamra et al. [39]. The garlic methanolic extract reduced the methane production without impairing the ruminal enzyme activity and *in vitro* DM degradability.

According to Lowry and Kennedy [37], quercetin, a phenolic aglycone, although insoluble in water, can be rapidly degraded by rumen microbes and enhance the rumen microbial activity. Lowry and Kennedy have also observed an inhibition of rumen microbial activity in the presence of catechin, despite of its close structural relationship to quercetin. These observations are comparable with the results obtained in this experiment showing the positive effects of quercetin and negative effects of catechin on rumen microbial activities.

The adenine, guanine, and purine content of control group were 2.1, 1.4, and 3.6 *μ*moL, respectively (Table 5). The addition of naringin and quercetin did not affect these values significantly; whereas the adenine, guanine, and purine content were significantly (*P* < 0.05) decreased upon addition of flavone, myricetin, catechin, rutin, and kaempferol. The estimated EMPS values of control were 0.10 *μ*moL purine/mL gas and 0.08 *μ*moL purine/mmoL total VFA and these values did not show significant difference when compared to both naringin and quercetin treated samples. However, flavone, myricetin, catechin, rutin, and kaempferol significantly (*P* < 0.05) decreased the EMPS when compared to the control. Similarly, these parameters supported the results obtained in DM degradability, total gas production, total VFAs, and enzyme activities of naringin- and quercetin-treated samples.

Broudiscou et al. [40] reported that the *A. millefolium, A. chamissonis,* and *L. angustifolia* leaves extracts which contained flavonoids increased without changes or decreased the EMPS, respectively. The variations in the results may relate to the type and concentration of the flavonoids present in the plant extract. In case of high concentration of flavonoids, the EMPS may decrease as observed in this study. Flavonoids used in this study were capable of modulating the EMPS; however, the appropriate levels to increase the EMPS need to be investigated. 

The precision of rumen microbial quantification using real-time PCR is revealed by the slope of standard curve and the PCR amplification efficiency values (Table 6). The slope and amplification efficiency obtained in this research ranged from −3.30 to −3.43 and from 95.6 to 102.8, respectively. Zhang and Fang [41] recommended the reliable standard curve in practice to have slope between −3.0 and −3.9 corresponding to PCR efficiencies of 80–115%. Thus, all the values for the slope and PCR amplification efficiency obtained in this study were in the acceptable range. 

The quantity of the rumen microbes affected by flavonoids is presented in Table 7. As observed with other parameters, the addition of naringin and quercetin had no significant effects on the population of general bacteria, general fungi, *Fibrobacter succinogenes, Ruminococcus albus, *and *Ruminococcus flavefaciens* when compared to the control. While these flavonoids significantly (*P* < 0.05) suppressed the population of total protozoa and total methanogens. The addition of flavone, myricetin, catechin, rutin, and kaempferol significantly (*P* < 0.05) reduced the population of almost all of the rumen microorganisms. The reduction of methane producing microorganisms is reflective of the decrease in methane production as shown in Table 2. It has been suggested that the flavonoids directly [1] or through new derivatives produced upon biotransformation or degradation [42] affect the rumen microbial activity. The effects of naringin and quercetin towards rumen microbes are desirable and they should be considered as alternative compounds to manipulate the rumen microbes towards maintaining the cellulolytic bacteria with lower protozoa and methanogens population.

The flavonoids generally act against microorganisms through inhibition of cytoplasmic membrane function, inhibition of bacterial cell wall synthesis, or inhibition of nucleic acid synthesis [34]. In addition, the antimicrobial potential of flavonoids is dependent on the number and the position of hydroxyl groups and presence of aliphatic and glycosyl groups in their structures. For instance, the active flavonoids against Methicillin-resistant *Staphylococcus aureus* are hydroxyl group at position 5 of flavones and flavanones [43]. Moreover, Mirzoeva et al. [44] reported the antibacterial action of quercetin and naringin against *E. coli* through disruption of proton motive force and inhibition of bacterial motility. To date, no much information is available on the mechanism of action of flavonoids against rumen microbes. The results obtained in this study indicated that flavone, myricetin, and kaempferol markedly reduced rumen microbial fermentation activity while catechin and rutin showed minimal effect. In contrast, naringin and quercetin maintained rumen microbial fermentation activity, with significant reduction in methane production.

## 4. Conclusions

The naringin and quercetin at the concentration of 4.5% (w/w) of the substrate (on dry matter basis) suppressed methane production and decreased rumen protozoa and methanogens population. The DM degradability and other fermentation parameters were not affected by these flavanoids. Future studies on feeding ruminant with plants rich in quercetin and naringin may allow the development of a natural and acceptable technique to manipulate rumen fermentation towards lower methane production.

## Figures and Tables

**Table 1:** PCR primer sets used in this study*.

Microorganism	Forward	Reverse	Amplicon size (bp)	Reference
General bacteria	cggcaacgagcgcaaccc	ccattgtagcacgtgtgtagcc	130	[45]
General fungi	gaggaagtaaaagtcgtaacaaggtttc	caaattcacaaagggtaggatgatt	120	[45]
Total protozoa	gctttcgwtggtagtgtatt	cttgccctcyaatcgtwct	223	[46]
Total methanogens	cgwagggaagctgttaagt	taccgtcgtccactcctt	343	[47]
*Ruminococcus albus *	ccctaaaagcagtcttagttcg	cctccttgcggttagaaca	175	[48]
*Ruminococcus flavefaciens *	cgaacggagataatttgagtttacttagg	cggtctctgtatgttatgaggtattacc	132	[45]
*Fibrobacter succinogenes *	gttcggaattactgggcgtaaa	cgcctgcccctgaactatc	121	[45]

*Primer sequence (5′→3′).

**Table 2:** Effects of flavonoids on dry matter degradability, total gas, methane and gas production parameters.

Items	Treatments								SEM
	Ctrl	F	M	N	C	R	Q	K	
Dry matter degradability (%)	87.9^a^	81.3^b^	82.1^b^	86.1^a^	82.0^b^	81.5^b^	85.6^a^	83.2^b^	1.2
Total gas (mL/24 h)	36.1^c^	28.1^d^	30.6^d^	47.8^a^	36.9^c^	40.9^b^	43.0^b^	34.8^c^	0.94
CH_4_ (mL/g DM)	8.6^a^	5.7^cd^	4.9^d^	6.3^c^	7.9^ab^	7.2^b^	6.2^c^	5.3^d^	0.29
(*a* + *b*) (mL)	41.1^c^	32.4^d^	34.2^d^	56.7^a^	43.7^c^	48.7^b^	55.4^a^	40.3^c^	1.63
*c* (h^−1^)	0.08^ab^	0.09^a^	0.09^a^	0.05^b^	0.09^a^	0.06^b^	0.06^b^	0.09^a^	0.007

Ctrl: control F: flavone, M: myricetin, N: naringin, C: catechin, R: rutin, Q: quercetin, and K: kaempferol.

*a*, *b*, *c*, and *a* + *b* are calculated from the exponential equation *p* = *a* + *b*(1 − *e* ^*ct*^).

(*a* + *b*) = potential extent of gas production, *c* = gas production rate constant for the insoluble fraction (*b*).

Means within the same row with different superscripts are significantly different (*P* < 0.05).

**Table 3:** Effects of flavonoids on pH, ammonia, and volatile fatty acids.

Items	Treatments								SEM
	Ctrl	F	M	N	C	R	Q	K	
pH	6.8	6.8	6.8	6.8	6.8	6.8	6.8	6.8	0.01
Ammonia N (mg/100 mL)	36.5	37.6	37.5	36.2	36.2	36.2	37.4	35.6	0.57
Total VFA (mM)	47.3^a^	41.3^b^	39.1^c^	47.0^a^	47.1^a^	46.7^a^	46.5^a^	42.3^b^	0.58
Acetic acid (molar %)	58.0^a^	52.6^b^	53.3^b^	60.2^a^	58.7^a^	60.1^a^	60.5^a^	53.6^b^	1.71
Propionic acid (molar %)	19.5^a^	16.2^b^	16.9^b^	17.8^ab^	19.0^a^	17.5^ab^	17.6^ab^	16.6^b^	0.75
Butyric acid (molar %)	13.8^b^	17.4^a^	18.1^a^	15.1^b^	15.5^b^	15.3^b^	15.1^b^	17.7^a^	0.66
C2 : C3 ratio^b^	3.0^b^	3.2^ab^	3.1^ab^	3.4^a^	3.1^ab^	3.4^a^	3.4^a^	3.2^ab^	0.20

Ctrl: control F: flavone, M: myricetin, N: naringin, C: catechin, R: rutin, Q: quercetin, and K: kaempferol.

C2 : C3: acetate : propionate ratio.

Means within the same row with different superscripts are significantly different (*P* < 0.05).

**Table 4:** Effects of flavonoids on the specific activity of enzymes in buffered rumen fluid.

Enzymes (*μ*mol/min/mg protein)	Treatments								S.E.M
	Ctrl	F	M	N	C	R	Q	K	
CMCase	0.45^a^	0.31^bc^	0.28^c^	0.43^a^	0.35^b^	0.34^b^	0.41^ab^	0.29^c^	0.05
FPase	0.29^a^	0.15^c^	0.14^c^	0.28^a^	0.22^b^	0.23^b^	0.27^a^	0.14^c^	0.03
Xylanase	0.82^a^	0.47^b^	0.41^b^	0.76^a^	0.52^b^	0.53^b^	0.75^a^	0.42^b^	0.11
*β*-Glucosidase	0.14^a^	0.07^b^	0.08^b^	0.15^a^	0.09^b^	0.09^b^	0.13^a^	0.08^b^	0.006

Ctrl: control F: flavone, M: myricetin, N: naringin, C: catechin, R: rutin, Q: quercetin, and K: kaempferol.

Means within the same row with different superscripts are significantly different (*P* < 0.05).

**Table 5:** Effects of flavonoids on purine content and efficiency of rumen microbial protein synthesis.

	Treatments								SEM
	Ctrl	F	M	N	C	R	Q	K	
Adenine (*μ*mol)	2.1^a^	1.3^c^	1.3^c^	2.2^a^	1.4^bc^	1.5^bc^	2.0^a^	1.3^c^	0.07
Guanine (*μ*mol)	1.4^a^	0.9^b^	1.0^b^	1.4^a^	1.0^b^	1.0^b^	1.3^a^	0.9^b^	0.07
Purines (*μ*mol)	3.6^a^	2.2^c^	2.3^c^	3.7^a^	2.4^bc^	2.6^bc^	3.4^a^	2.2^c^	0.14
Efficiency of microbial protein synthesis (EMPS)									
*μ*mol purine/mL gas	0.10^a^	0.07^b^	0.07^b^	0.08^ab^	0.06^b^	0.06^b^	0.08^ab^	0.06^b^	0.01
*μ*mol purine/mmol total VFA	0.08^a^	0.05^b^	0.06^b^	0.08^a^	0.05^b^	0.05^b^	0.08^a^	0.05^b^	0.01

Ctrl: control F: flavone, M: myricetin, N: naringin, C: catechin, R: rutin, Q: quercetin, and K: kaempferol.

Means within the same row with different superscripts are significantly different (*P* < 0.05).

**Table 6:** The slope of the standard curve and real-time PCR amplification efficiency.

Microorganisms	Slope	Efficiency
General bacteria	−3.32	100.1
General fungi	−3.43	95.6
Total protozoa	−3.32	102.5
Total methanogens	−3.33	101.1
*Fibrobacter succinogenes *	−3.31	102.8
*Ruminococcus albus *	−3.30	100.9
*Ruminococcus flavefaciens *	−3.33	99.8

**Table 7:** Effect of flavonoids on different rumen microbial population.

Items	Treatments								SEM
	Ctrl	F	M	N	C	R	Q	K	
General bacteria × 10^14^ copies/mL of rumen fluid									
	6.5^a^	3.7^b^	3.5^b^	5.4^a^	5.3^a^	4.9^ab^	5.3^a^	3.4^b^	1.22

General fungi × 10^5^ copies/mL of rumen fluid									
	3.7^a^	2.1^b^	2.1^b^	3.2^a^	2.6^ab^	2.9^ab^	3.4^a^	2.3^b^	0.36

Total protozoa × 10^6^ copies/mL of rumen fluid									
	3.8^a^	1.1^c^	1.9^b^	1.9^b^	2.1^b^	2.6^ab^	2.3^b^	1.5^bc^	0.31

Total methanogens × 10^7^ copies/mL of rumen fluid									
	1.7^a^	1.0^b^	0.7^b^	0.6^b^	1.1^ab^	1.3^a^	0.9^b^	1.1^ab^	0.22

*Fibrobacter succinogenes* × 10^6^ copies/mL of rumen fluid									
	3.5^a^	1.4^c^	1.6^bc^	3.2^a^	2.7^ab^	2.5^b^	3.1^a^	1.4^c^	0.26

*Ruminococcus albus* × 10^5^ copies/mL of rumen fluid									
	2.4^a^	1.5^bc^	1.8^b^	2.3^a^	2.0^ab^	1.8^b^	2.4^a^	1.5^bc^	0.18

*Ruminococcus flavefaciens* × 10^5^ copies/mL of rumen fluid									
	5.1^a^	3.7^b^	3.2^bc^	5.2^a^	4.2^b^	4.3^ab^	4.9^a^	3.1^c^	0.28

Ctrl: control F: flavone, M: myricetin, N: naringin, C: catechin, R: rutin, Q: quercetin, and K: kaempferol.

Means within the same row with different superscripts are significantly different (*P* < 0.05).
